# Transwell Culture with Adipose Tissue-Derived Stem Cells and Fertilized Eggs Mimics the In Vivo Development of Fertilized Eggs to Blastocysts in the Fallopian Tube: An Animal Study

**DOI:** 10.3390/antiox13060704

**Published:** 2024-06-08

**Authors:** Toyofumi Hirakawa, Kazuhiko Nakabayashi, Noriko Ito, Kenichiro Hata, Shiori Imi, Mami Shibata, Daichi Urushiyama, Kohei Miyata, Fusanori Yotsumoto, Shin’ichiro Yasunaga, Tsukasa Baba, Shingo Miyamoto

**Affiliations:** 1 Department of Obstetrics & Gynecology, Faculty of Medicine, Fukuoka University, Fukuoka 814-0180, Japan; thira@fukuoka-u.ac.jp (T.H.); imi.shiori@fukuoka-u.ac.jp (S.I.); shibatamm@fukuoka-u.ac.jp (M.S.); duru@fukuoka-u.ac.jp (D.U.); kmiyata@outlook.com (K.M.); yotsumoto@cis.fukuoka-u.ac.jp (F.Y.); 2 Department of Maternal-Fetal Biology, National Research Institute for Child Health and Development, Tokyo 157-8535, Japan; nakabaya-k@ncchd.go.jp (K.N.); ito-nor@ncchd.go.jp (N.I.); hata-k@ncchd.go.jp (K.H.); 3 Department of Biochemistry, Faculty of Medicine, Fukuoka University, Fukuoka 814-0180, Japan; syasunag@fukuoka-u.ac.jp; 4 Department of Obstetrics & Gynecology, School of Medicine, Iwate Medical University, Morioka 028-3694, Japan; babatsukasa@gmail.com; 5 Cybele Corporation Limited, 2-128-14 Sugukita, Kasugashi 816-0864, Japan

**Keywords:** blastocyst, extracellular vesicles, adipose stem cells, stemness, Transwell culture

## Abstract

Many countries, including Japan, are experiencing declining birth rates. Assisted reproductive technologies have consistently demonstrated good results in resolving infertility. Although the development of fertilized eggs into blastocysts has been recognized as a crucial step in assisted reproductive technologies, the involved mechanisms are currently unclear. Here, we established a new culture system for the in vitro development of fertilized eggs into blastocysts. In the Transwell culture system, the rate of blastocysts hatching from fertilized eggs cultured with adipose-derived stem cells (ASCs) was significantly higher than that of blastocysts cultured only with fertilized eggs. Gene ontology analysis revealed that the developed blastocysts displayed essential gene expression patterns in mature blastocysts. Additionally, when cultured with 3rd-passage ASCs, the developed blastocysts expressed the core genes for blastocyst maturation and antioxidant properties compared to those cultured only with fertilized eggs or cultured with 20th-passage ASCs. These results suggest that the Transwell culture system may imitate the in vivo tubal culture state for fertilized eggs. Exosomes derived from stem cells with stemness potential play a powerful role in the development of blastocysts from fertilized eggs. Additionally, the exosomes expressed specific microRNAs; therefore, the Transwell culture system resulted in a higher rate of pregnancy. In future, the extraction of their own extracellular vesicles from the culture medium might contribute to the development of novel assisted reproductive technologies.

## 1. Introduction

Approximately 15–20% of couples worldwide have been diagnosed with infertility or sterility, which is defined as the failure to conceive despite ordinary sexual intercourse without contraception [1]. Assisted reproductive technologies (ARTs) have contributed to solving medical, economic, and social issues related to infertility [2]. The use of ARTs to overcome infertility has been associated with improvements in the in vitro environment for embryo production [3]. Although various molecules in the female reproductive system are involved in the equilibrium between oxidants and antioxidants, the risk of oxidative damage is possibly magnified in an in vitro system of ART [4]. ARTs involve many artificial procedures that may cause damage, such as oxidative stress [5]. Consequently, many reactive oxygen species (ROS) are generated and accumulate in in vitro-produced embryos during the process of ART. Oxidative damage results in the impairment of blastocyst development and hatching, leading to a decrease in the fertilization rate [6,7,8]. Accumulating evidence has revealed that antioxidants play a pivotal role in reducing oxidative damage in assisted reproductive technologies, particularly in in vitro-produced embryos [9]. The addition of antioxidants activates the development of embryos during the preimplantation process. Many studies have shown that the development of blastocysts from fertilized eggs, as well as the preimplantation of blastocytes, can be improved by the addition of antioxidant substances to the culture medium of fertilized eggs [10,11,12,13]. Recent studies have indicated that mesenchymal stem cells possess antioxidant properties against biological events [14]. Furthermore, a few studies have reported that mesenchymal stem cells are associated with the development of blastocysts in in vitro-produced embryos, suggesting that certain substances secreted from mesenchymal stem cells may play a critical role in the development of blastocysts [8,15]. In principle, stem cells can self-renew and differentiate into a variety of specialized cell types possessing infinite regenerative potential through their trophic properties [16,17,18,19,20]. Stem cells originate from four main sources: embryonic, fetal, adult, and differentiated somatic cells [21]. Mesenchymal stem cells isolated from differentiated somatic cells have been used in regenerative medicine [22]. They also play an influential role in the regeneration of damaged cells and tissues in various diseases by secreting paracrine factors and extracellular vesicles (EVs) [23]. To date, EVs secreted from stem cells have attracted considerable attention due to their ability to retain stem cell stemness. Adipose-derived mesenchymal stem cells (ASCs) are commonly used in various clinical fields because of their strong antioxidant properties [24,25,26,27]. Among the different types of MSCs, ASCs are unique owing to their minimally invasive harvesting procedure, capacity for efficient bulk retrieval, and commendable cell proliferation rates [28]. In addition, EVs derived from mesenchymal stem cells have been recognized as promising therapeutic agents for various disorders, serving as antioxidant molecules [29,30]. Any cell type can secrete EVs, both in physiological and pathological conditions. In principle, EVs can be broadly categorized into exosomes and ectosomes [31]. Ectosomes, which pinch off the surface of the plasma membrane via outward budding, consist of microvesicles, microparticles, and large vesicles with diameters of 50–1000 nm. Exosomes, which originate from endosomes, range from 40 to 160 nm in diameter. EVs contain many cellular components, including microRNAs, RNA, DNA, lipids, metabolites, cytokines, and proteins. Recently, EVs have emerged as an attractive research field because of their central role in intercellular communication. In female reproduction, EVs communicate important information between cells and tissues during folliculogenesis, fertilization, embryo formation, and implantation [32,33,34,35]. EVs derived from oviduct fluid may enhance embryo development [33]. However, it is not clear whether this positive effect can be attributed to these EVs. To date, a few reports have described that mesenchymal stem cells stimulate the development of blastocysts in in vitro-produced embryos, suggesting that a variety of paracrine factors as well as EVs may participate in the development of blastocysts in in vitro-produced embryos [8,15]. In this study, we aimed to examine the development of blastocysts from in vitro-produced embryos to create a new Transwell culture system for in vitro fertilization. In addition, we aimed to identify the paracrine factors and EVs secreted by mesenchymal stem cells that are involved in the development of blastocysts in in vitro-produced embryos. Embryos developed in Transwell culture showed enhanced expression of genes involved in cytoskeleton, pluripotency, and embryonic organ development. Furthermore, EVs derived from 3rd-passage stem cells possessed much more potential than those derived from 20th-passage stem cells did. EVs derived from 3rd-passaged stem cells contained various specific miRNAs.

## 2. Materials and Methods

### 2.1. Cell Culture

In a Transwell culture system, two different cell types can be cultured separately in each medium. By utilizing a 400 nm pore filter, specific substances present in the medium of the upper layer can be transferred to the medium of the lower layer.

### 2.2. Animals

Twelve-week-old ICR female mice were purchased from KBT Oriental (Saga, Japan) to obtain adipose tissue-derived stem cells (ASCs). Frozen 2-cell embryos were purchased from the Biosafety Research Center, Inc. (Hyogo, Japan). All animal experiments were approved by the Animal Experiment Committee of Fukuoka University (approval No. 1915132).

### 2.3. Isolation of ASCs

ASC isolation was conducted in accordance with a previously documented protocol [36]. In brief, subcutaneous adipose tissue specimens were obtained from ICR mice to procure adipose-derived regenerative cells (ADRCs). The ADRCs were cultivated in a MEMα/GlutaMax medium (Gibco, Grand Island, NY, USA). Moreover, they were supplemented with 10% Exo-FBS (System Biosciences, Palo Alto, CA, USA) and a 1% antibiotic–antimycotic solution (Gibco) within a humidified incubator set at 37 °C with 5% CO_2_. For experimental purposes, ASCs from early or late passages (3rd or 20th passages, respectively) were utilized.

### 2.4. In Vitro Culture of Embryos

**Figure 1:**
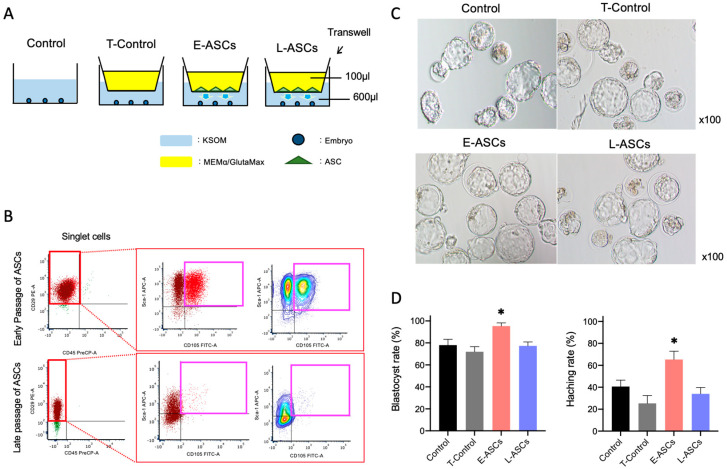
(**A**) Scheme of in vitro culture of embryos using a Transwell. Control: embryos incubated KSOM without Transwell; T-Control: no cells in the upper chamber; E-ASCs (adipose tissue-derived mesenchymal stem cells): early passage of ASCs (P3: 3rd passage) seeded in the upper chamber; L-ASCs: late passage of ASCs (P20: 20th passage) seeded in the upper chamber. (**B**) Multicolor flow cytometry analysis of cells in the plot of singlet cells based on staining for CD105, CD29, Sca-1, and CD45. The percentages of mesenchymal stem cells, including early passage (3rd passage) adipose tissue-derived stem cells (ASCs) and late passage (20th passage) ASCs, harboring the cell-surface expression markers CD29, Sca-1, and CD105, and negative CD45 are shown. Cells in pink box indicate mesenchymal stem cells. (**C**) Embryo morphology on Day 4. (**D**) Blastocyst and hatching rates of in vitro-produced mouse embryos co-cultured with ASCs. The mean percentage was calculated from three independent experiments. *n* = 150 per group. Data are presented as the mean ± standard error. * *p* < 0.05 vs. Control.

We divided the upper and lower chambers and used Transwell filters (Greiner Bio-One, 24-well, pore size 0.4 μm) for nanoparticle transport studies. ASCs were seeded into the upper chamber at a density of 1 × 10^5^ cells/well as superscript with 100 μL of MEMα/GlutaMax medium containing 10% Exo-FBS and 1% antibiotic–antimycotic solution. Thereafter, frozen 2-cell embryos were incubated in the lower chamber filled with 600 µL KSOM at 37 °C and 5% CO_2_ on Day 0 (Figure 1A). Both chambers underwent medium exchange on Day 2. We observed blastocyst formation and hatching on Day 4. Frozen 2-cell embryos incubated with KSOM without the Transwell filter were used as controls. No cells were observed in the upper chamber, which was defined as the T-control group. Early passage ASCs and late passage ASCs seeded in the upper chamber were defined as E-ASC and L-ASC groups, respectively (Figure 1A). Morphological evaluation of the mouse embryos was performed stepwise from the 2-cell stage to hatching.

### 2.5. Flow Cytometric Analyses

Flow cytometric analyses of ASCs were performed according to a previously reported protocol [36]. In brief, ASCs were suspended in a staining buffer (R&D Systems, Minneapolis, MN, USA). Following this, they were incubated with CD105, CD29, Sca-1, and CD45 (all from the Mouse Mesenchymal Stem Cell [MSC] Multi-Color Flow Kit, cat. no. FMC003; R&D Systems). Flow cytometry was performed using FACSAria^TM^ (Becton, Dickinson and Company, Franklin Lakes, NJ, USA).

### 2.6. Enzyme-Linked Immunosorbent Assay (ELISA)

Cell culture supernatant interleukin (IL)-6, vascular endothelial growth factor (VEGF)-A, leptin, adiponectin, and fibroblast growth factor (FGF)2 levels were determined by ELISA, following the manufacturer’s instructions. Mouse IL-6, VEGF-A, leptin, adiponectin, and FGF2 ELISA kits were purchased from Proteintech (Rosemont, IL, USA).

### 2.7. In Vitro Culture of Embryos with Antibody

Mouse VEGF Antibody (#AF-493-NA), Mouse IL-6 Antibody (#MAB406-100), Normal Rat IgG Control (#6-001-A), and Normal Goat IgG Control (#AB-108-Cmouse) were purchased from R&D Systems (Minneapolis, MN, USA). In a Transwell culture system with E-ASCs, these antibodies were suspended at 1 μg/mL in the KSOM at the lower chamber. We observed blastocyst formation and hatching on Day 4.

### 2.8. Generation of Stably Transfected ASCs

Short interfering RNAs (siRNAs) of the Universal Negative Control (#SIC001), IL-6 (#EMU011231), and VEGFA (#EMU062931) were purchased from Sigma-Aldrich Japan (Tokyo, Japan). The siRNAs were transfected into E-ASCs using Lipofectamine 2000 (Invitrogen). At 48 h post-transfection, the transfected E-ASCs were used for the in vitro culture of embryos using a Transwell. We observed blastocyst formation and hatching on Day 4. Supernatants in the lower chamber were harvested, and the protein levels of IL-6 and VEGFA were evaluated using an ELISA.

### 2.9. Ultracentrifugation to Isolate EVs

We isolated EVs from the supernatants of ASCs by ultracentrifugation, as described previously [37]. The supernatant from the lower chamber was harvested and centrifuged using a KUBOTA Centrifuge (Tokyo, Japan) at 300× *g* at 4 °C for 10 min to remove detached cells. Next, the supernatant was collected and centrifuged at 2000× *g* at 4 °C for 10 min to remove detached cells. Furthermore, the supernatant was collected and centrifuged at 10,000× *g* at 4 °C for 30 min to remove contaminating apoptotic bodies, microvesicles, and cell debris. Clarified cell culture medium was then centrifuged in a Beckman Coulter Optima™ MAX- Ultracentrifuge at 100,000× *g*_avg_ at 4 °C for 70 min with a Type MLS-50 rotor (k-factor: 71) to pellet the exosomes. The supernatant was removed carefully, and the crude exosome-containing pellets were resuspended in 1 mL of ice-cold phosphate-buffered saline (PBS) and pooled. A second round of ultracentrifugation [100,000× *g*_avg_ at 4 °C for 70 min with a Type MLS-50 rotor (k-factor: 71)] was carried out, and the resulting exosome pellet was resuspended in proper culture medium.

### 2.10. Nanoparticle Tracking Analysis (NTA) Measurement

Supernatants in the lower chamber were harvested, and nuclei and cell debris were removed by centrifugation. The number of nanoparticles (NPs) in the supernatant was measured by NTA using a NanoSight LM10 (NanoSight, Malvern, UK). The ideal measurement concentrations were determined by pretesting the ideal particle-per-frame value (20–100 particles/frame). The following settings were set according to the manufacturer’s software manual (NanoSight LM10 User Manual, MAN0510-04-EN-00). The camera level was adjusted to level 8 until all particles were distinctly visible and did not exceed a particle signal saturation of over 20%. The autofocus was adjusted to avoid indistinct particles. After capturing, the videos were analyzed using NanoSight Software NTA 3.0.

### 2.11. Western Blot Analysis

EV pellets obtained by ultracentrifugation were resuspended in RIPA Lysis and Extraction Buffer (#89900; ThermoFisher Scientific, Tokyo, Japan) supplemented with Protease Inhibitor Cocktail (#78410; ThermoFisher Scientific) and Phosphatase Inhibitor Cocktail (#78420; ThermoFisher Scientific). The total proteins of EVs were resolved by 10% precast SDS/PAGE gels (#195-14951; FUJIFILM Wako, Osaka, Japan) and transferred to a Poly Vinylidene DiFluoride (PVDF) membrane using iBlot2 (ThermoFisher Scientific). The membrane was blocked with PVDF Blocking Reagent for Can Get Signal^®^ (TOYOBO, Osaka, Japan) for 1 h at room temperature under constant shaking. Subsequently, the membranes were incubated with rabbit polyclonal CD63 (1:1000, Proteintech), CD9 (1:1000, Cell Signaling Technology, Danvers, MA, USA), and CD81 (1:1000, Cell Signaling Technology) antibodies overnight at 4 °C. Membranes were washed three times with 1× Tris-buffered saline containing Tween 20 (TBS-T) and then incubated with rabbit horseradish peroxidase-conjugated secondary antibodies (#7074S; Cell Signaling Technology; 1:1000 dilution) in a blocking solution for 1 h at room temperature under constant shaking. After washing the membranes thrice with TBS-T, the blots were developed using Immobilon Western Chemiluminescent HRP Substrate (#61-0205-40; Merck Millipore, Burlington, MA, USA) and visualized using an Amersham^TM^ Imager 680 Imaging System (Cytiva, Tokyo, Japan) and Amersham^TM^ Imager 680 Analysis Software Version 2.0 (Cytiva, Tokyo, Japan).

### 2.12. Extracellular Nanovesicles Labeling and Uptake

The EVs obtained via ultracentrifugation were labeled using the ExoGlow-Membrane EV Labeling Kit (Cat # EXOGP400A-1, System Biosciences) as per the manufacturer’s protocol. Following labeling, the EVs were suspended in KSOM and introduced to morulae, which were maintained at 37 °C in a humidified atmosphere containing 5% CO_2_. Prior to cell treatment, all samples underwent ultracentrifugation. Moreover, any residual dye from the exosome labeling process was eliminated using a PD MiniTrap G-25 (Cytiva) in accordance with the manufacturer’s instructions. Subsequently, the labeled exosomes were administered after a 30 min incubation with Pitstop2. Following a 24 h incubation period, the uptake process was halted by the washing and fixation of the cells in a 3.7% PFA solution for 10 min. Subsequent staining with DAPI allowed for the visualization of the cells under a fluorescence microscope (Keyence, Osaka, Japan).

### 2.13. Library Preparation and Sequencing of EV miRNAs

To obtain the supernatant of E-ACSs or L-ASCs, ASCs were incubated on a 100 mm plate in 10 mL of MEMα/GlutaMax medium containing 10% Exo-FBS and 1% antibiotic-antimycotic at 37 °C and 5% CO_2_. The supernatant was collected after 48 h and filtered using a 0.22 μm filter to remove detached cells and debris (N = 3). For miRNA-seq, miRNAs were obtained from the supernatant using the exoRNeasy Serum/Plasma Maxi Kit (QIAGEN). Sequencing libraries were constructed using a QIAseq miRNA Library Kit (QIAGEN, Valencia, CA, USA) according to the manufacturer’s protocol. Library quality was assessed using the Agilent 2100 Bioanalyzer High Sensitivity DNA Kit (Agilent Technologies, Santa Clara, CA, USA). The pooled libraries of the samples were sequenced using NextSeq 500 (Illumina, Inc., San Diego, CA, USA) in 76 base-pair (bp) single-end reads. Raw data were applied to the bioinformatics pipeline, which included quality control with FastQC ver.0.12.1 and MultiQC ver.1.18.

### 2.14. Quantifying the miRNA Expression Level and Detection of Differentially Expressed Genes

The QIAseq miRNA Library Kit adopts a unique molecular indices (UMIs) system, enabling the unbiased and accurate quantification of mature miRNAs. All reads assigned to a particular miRNA (miRBase Release 22) were counted, and the associated UMIs were aggregated to count unique molecules using CLC Genomics Workbench 23.0.2 (QIAGEN, Valencia, CA, USA). The UMI counts of miRNAs were normalized using CPM (TMM-adjusted), which stands for Trimmed Mean of M values adjusted Counts Per Million [38], in the CLC Genomics Workbench. Normalized values were subjected to downstream analyses using StrandNGS 4.0 software (Agilent Technologies, Santa Clara, CA, USA). Statistical analysis between two groups (“sample” and “control”) was performed by moderated *t*-test [39]. Differentially expressed genes (DEGs) were considered significant if the Fold Change (FC) was >1.5.

### 2.15. cDNA Library Preparation and RNA Sequencing of Blastocysts

Blastocyst samples were collected from the control, E-ASC, and L-ASC groups on Day 4 (N = 8 for each group). Sequencing libraries were prepared for each sample by using NEBNext^®^ Single Cell/Low Input RNA Library Prep Kit for Illumina^®^ (New England BioLabs, Ipswich, MA, USA) and NEBNext^®^ Multiplex Oligos for Illumina^®^ (96 Unique Dual Index Primer Pairs) (New England BioLabs) in accordance with the manufacturer’s instructions (Illumina, San Diego, CA, USA). Library quality was assessed using the Agilent 2100 Bioanalyzer/High Sensitivity (Agilent Technologies). The libraries were sequenced using Illumina’s sequencer HiSeq X (Illumina, San Diego, CA, USA) with paired-end 151 bp and dual index settings.

### 2.16. RNA-Sequencing Data Analysis of Blastocysts

Raw sequencing reads of 24 RNA sequencing libraries, ranging from 15.0 to 22.4 million read pairs, were subjected to transcript quantification using Salmon 1.5.2 (https://salmon.readthedocs.io/en/latest/salmon.html, accessed on 23 July 2021) [40]. To run the mapping-based mode for salmon, a salmon transcriptome index was generated for gencode.vM23.transcripts.fa.gz using the entire human genome (GRCm38.primary_assembly.genome.fa.gz) as the decoy sequence. The resultant quant.sf data of the 24 samples were integrated into one file using the R package tximport 1.20.0 [41]. The resultant count data for 54,281 gene IDs were subjected to further analyses using an R package, EdgeR 3.36.0 [42], such as filtering, normalization, clustering analyses, and the identification of DEGs between two conditions (FDR q-value < 0.0.5 as a threshold). Gene ontology analyses of the DEGs were conducted using Metascape 3.5 [43].

### 2.17. Statistical Analysis

All data were analyzed using the Prism 8 software (GraphPad Software Inc., San Diego, CA, USA). The results were shown as mean ± standard error. Data regarding blastocyst formation rates, hatching rates, cytokine concentrations, and nanoparticle concentrations among the different groups were analyzed using the Mann–Whitney U test and Benjamini–Hochberg method. *p* values were considered significant when they were less than 0.05.

## 3. Results

### 3.1. Cellular Characteristics of ASCs

To examine the expression of cell-surface markers of MSCs, flow cytometry was performed using antibodies against CD105, CD29, and Sca-1 as positive markers and CD45 as a negative marker. For cells in the P1, P2, and P3 plots, the phenotype was analyzed based on stained cell surface markers (Figure 1B). The percentage of MSCs in ASCs was 48.6 ± 4.3% (n = 3) at the early cell passage (passage 3) (Figure 1B), which decreased to 2.1 ± 0.4% (n = 3) by the late cell passage (passage 20) (Figure 1B). These results suggested that serial subcultivation induced a marked decrease in ASC stemness.

### 3.2. Effect of ASCs on Embryo Development

In the control group, 78.0 ± 7.5% of 2-cell embryos developed into a blastocyst on Day 4. In contrast, 95.3 ± 4.1% of the E-ASCs group developed into a blastocyst, and 25.3 ± 10.0% hatched (Figure 1C,D). In the E-ASC group, the embryo development and hatching rates were significantly higher than those in the control group (*p* < 0.05). However, no significant differences in development or hatching rates were observed among the control, T-control, and L-ASC groups (Figure 1D). These results indicate that the co-culture of E-ASCs with embryos using Transwell increased embryo development.

### 3.3. Effect of Cytokines on Embryo Development

**Figure 2:**
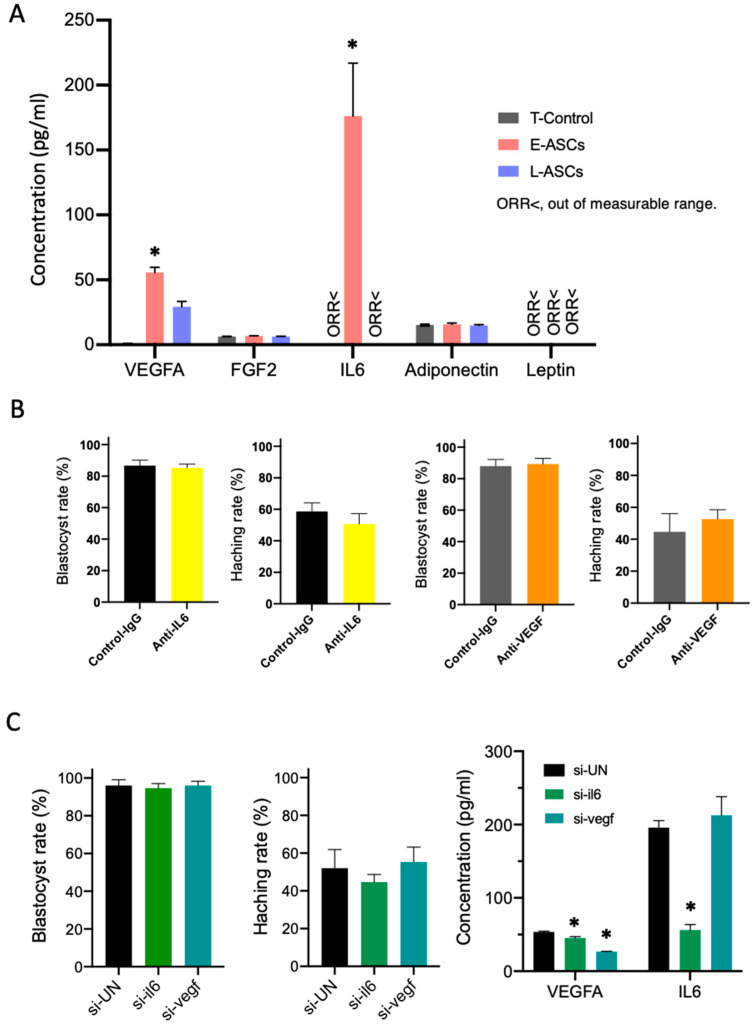
(**A**) Factor composition of the lower chamber. T-Control: no cells in the upper chamber; E- ASCs (adipose tissue-derived mesenchymal stem cells): the early passage of ASCs (P3: 3rd passage) seeded in the upper chamber; L-ASCs: the late passage of ASCs (P20: 20th passage) seeded in the upper chamber. Data are presented as the mean ± standard error. * *p* < 0.05 vs. T-Control. <ORR: out of measurable range. (**B**) Blastocyst and hatching rates for in vitro culture of embryos with mouse VEGF antibody or mouse IL-6 antibody. (**C**) Blastocyst rates, hatching rates, and concentration of VEGFA and IL-6 for in vitro culture of embryos with E-ASCs transfected with short interfering RNA (siRNA). Data are presented as the mean ± standard error. * *p* < 0.05 vs. Control. UN: Universal control.

In the E-ASC groups, IL-6 and VEGFA protein levels were significantly higher than those in the control and L-ASC groups (*p* < 0.05). Only the VEGFA protein levels of the L-ASC group were significantly higher than those of the control group (*p* < 0.05). No significant differences were detected in the protein levels of FGF2, leptin, and adiponectin among the T-control, E-ASC, and L-ASC groups (Figure 2A).

Blastocyst development and hatching rates did not decrease, even when anti-IL-6 antibodies or anti-VEGFA antibodies were added to the culture medium (Figure 2B).

In addition, the blastocyst differentiation ability was not reduced by E-ASC-transfected siRNA, which inhibited IL-6 and VEGFA secretion (Figure 2C).

### 3.4. Comparison of Exosome Abundance and miRNAs Contained by Different ASC Passages

**Figure 3:**
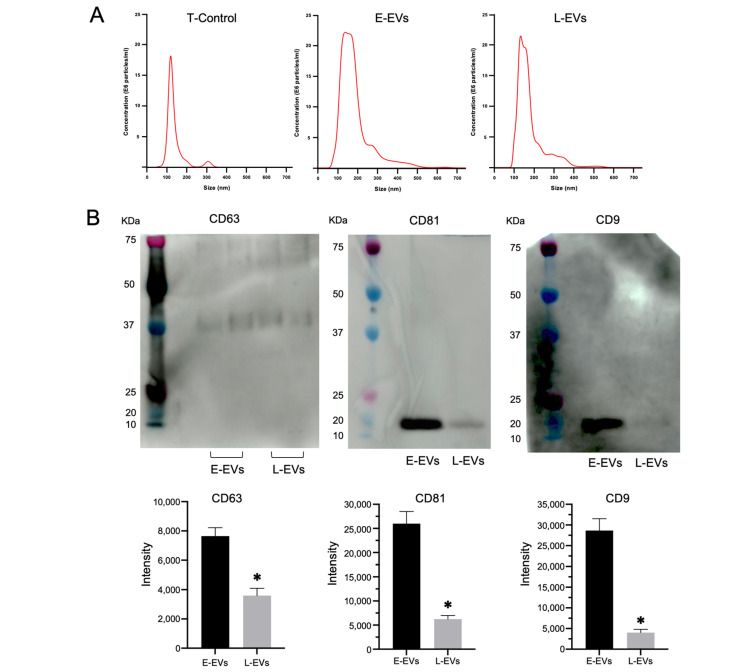

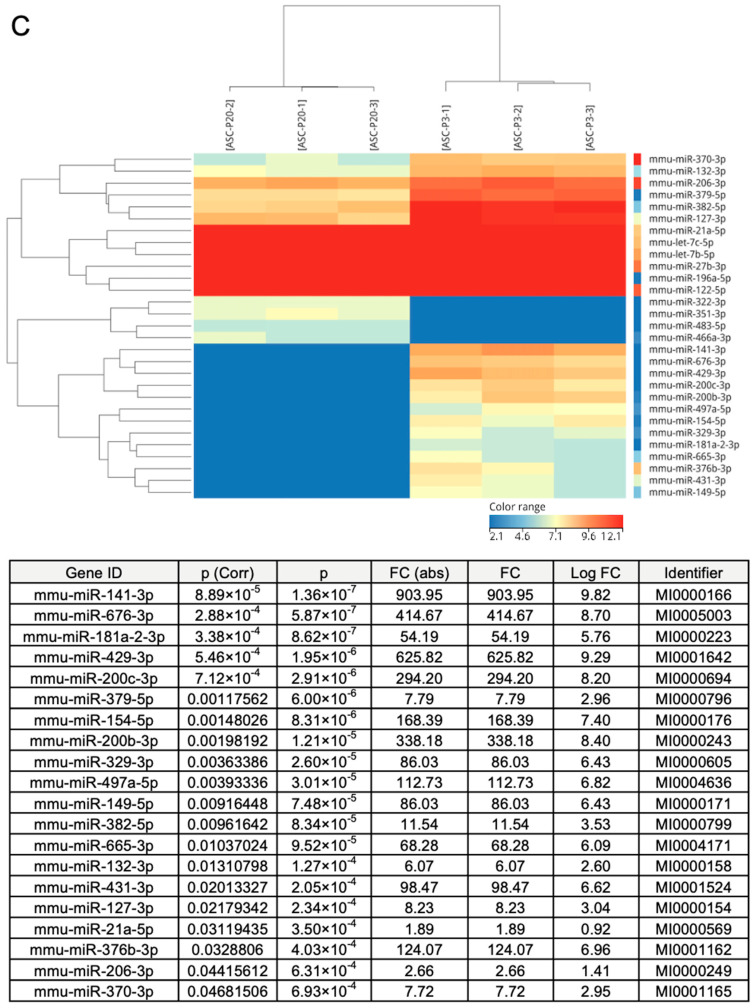

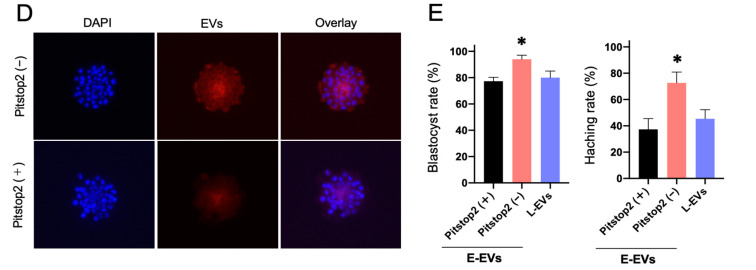
(**A**) The NanoSight instrument indicates the concentration or size of nanoparticles in the lower chamber. T-Control: no cells in the upper chamber; E-ASCs (adipose tissue-derived mesenchymal stem cells): the early passage of ASCs (P3: 3rd passage) seeded in the upper chamber; L-ASCs: late passage of ASCs (P20: 20th passage) seeded in the upper chamber. (**B**) Detection of exosomal markers CD63, CD81, and CD9 in exosomes isolated from the supernatant of E-ASCs or L-ASCs determined by Western blotting. * *p* < 0.05 vs. E-EVs. (**C**) Expression analysis of miRNAs in E-EVs and L-EVs determined by Next-Generation Sequencing. (**D**) Fluorescence microscopy showing the E-EVs taken up by the blastocyst. Nuclei were counterstained with 4′,6-diamidino-2-phenylindole (DAPI). (**E**) Blastocyst formation and hatching rates of in vitro-produced mouse embryos with exosomes of E-ASCs (E-EVs) or exosomes of L-ASCs (L-EVs). E-ASC group with Pitstop2 (+) or without Pitstop2 (–). Data are presented as the mean ± standard error. * *p* < 0.01 vs. E-EVs with Pitstop2.

EVs, including exosomes, shedding vesicles, prostasomes, and apoptotic bodies, are membrane vesicles of 40–1000 nm that are released from many cell types, including red blood cells, platelets, lymphocytes, dendritic cells, endothelial cells, and tumor cells [44]. In particular, CD9, CD63, and CD81 are recognized as exosome-specific markers [45]. In this study, the size of the nanoparticles ranged between 50 and 600 nm, and most of them were between 100 and 200 nm in all media groups. The numbers of nanoparticles were 8.2 × 10^8^ ± 3.51 × 10^7^/mL in the T-Control group, 2.74 × 10^9^ ± 1.90 × 10^8^/mL in the E-ASC group, and 1.98 × 10^9^ ± 2.75 × 10^8^/mL in the L-ASC group (Figure 3A). These nanoparticles expressed CD63, CD81, and CD9, as determined by Western blotting, and were presumed to be exosomes (Figure 3B). From the difference in the number of nanoparticles, the numbers of exosomes in E-ASCs (E-EVs) and L-ASCs (L-EVs) were estimated at approximately 1.92 × 10^9^/mL and 1.16 × 10^9^/mL, respectively. Expression analysis of microRNAs in EVs derived from E-ASCs and L-ASCs utilizing a next-generation sequencing technique unveiled an upregulation of miR-141-3p, miR-676-3p, miR-181a-2-3p, miR-429-3p, miR-200c-3p, miR-379-5p, miR-154-5p, miR-200b-3p, miR-329-3p, miR-497a-5p, miR-149-5p, miR-382-5p, miR-665-3p, miR-132-3p, miR-431-3p, miR-127-3p, miR-21a-5p, miR-376b-3p, miR-206-3p, and miR-370-3p (Figure 3C). These findings suggest that while both E-ASCs and L-ASCs release EVs, E-ASCs demonstrate a greater abundance and a distinct repertoire of miRNAs.

### 3.5. Effect of Exosomes on Embryo Development

The E-ASC-EVs fluorescently labeled red by the ExoGlow-Membrane EV Labeling Kit were observed to be integrated into the inner blastocyst, as verified through fluorescence microscopy. On the other hand, the inhibition of E-ASC-EV uptake by pitstop2 was similarly confirmed by fluorescence microscopy (Figure 3D). The blastocyst development and hatching rates when incubated in KSOM mixed with E-ASCs-EVs (94.0 ± 4.3% and 72.7 ± 11.6%, respectively) were higher than those of blastocysts with inhibited E-ASCs-EVs uptake by pitstop2 (77.3 ± 4.1% and 37.3 ± 11.6%, respectively) (Figure 3E). However, L-ASC-EVs did not improve the blastocyst formation rate (Figure 3E).

### 3.6. Effect of Exosomes on the Gene Expression of Blastocysts

**Figure 4:**
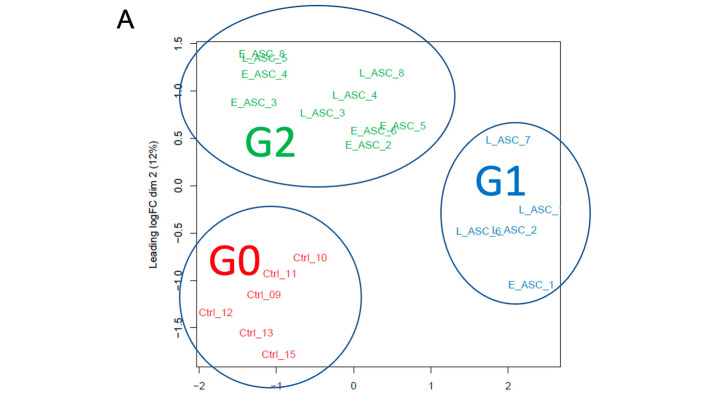

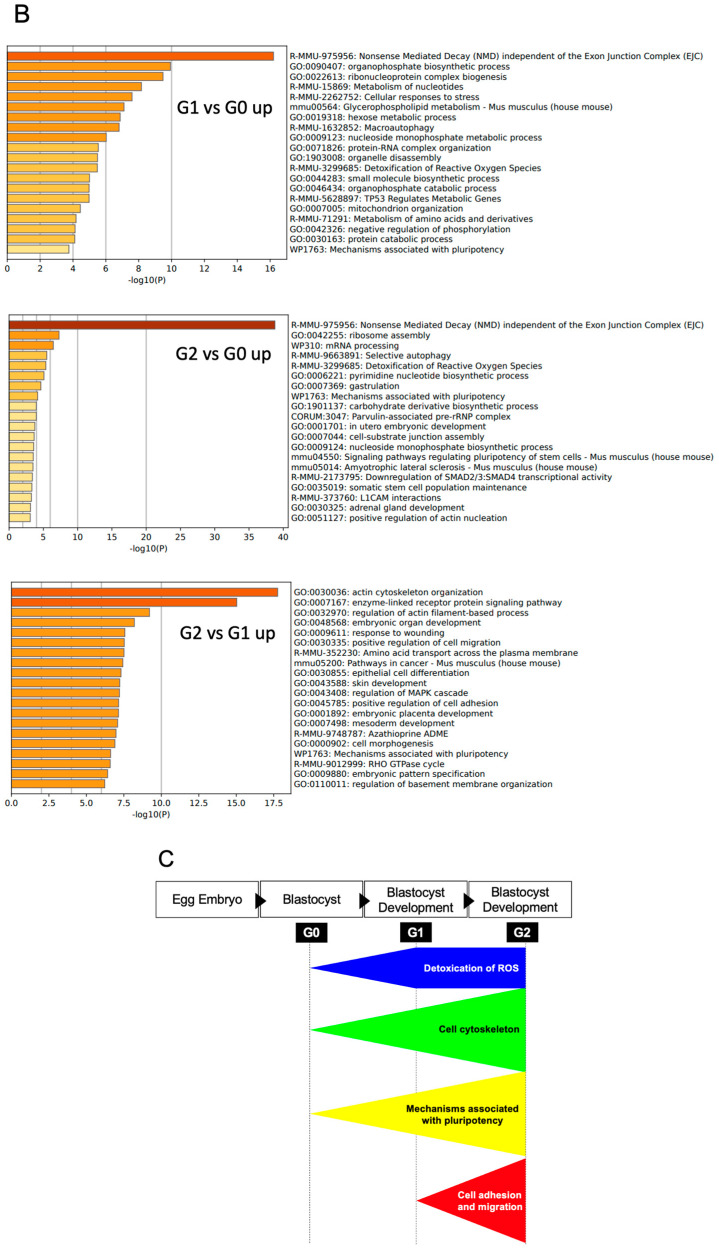
Effects of exosomes on the gene expression of blastocysts cultured in vitro. (**A**) Identification of three clusters based on transcriptome data. (**B**) Characterization of up-regulated genes affected by E-EVs and L-EVs. (**C**) Summary of the GO analysis among the three groups, G0, G1, and G2: the genes associated with the cell cytoskeleton, pluripotency, and detoxification of reactive oxidative stress were remarkably up-regulated in G2, compared with those in G0 and G1, and in G1, they also significantly increased, compared with those in G0. In addition, the genes associated with cell adhesin and migration in G2 indicated high expression compared with those in G1.

We conducted a transcriptome analysis of the blastocysts grown from the 2-cell embryos in the KSOM (control [Ctrl] N = 8), the KSOM supplemented with E-ASCs-EVs (N = 8), and the KSOM supplemented with L-ASCs-EVs (N = 8). Based on our initial evaluation of the transcriptome data of 24 blastocysts by hierarchical clustering and multidimensional scaling (MDS) using EdgeR, we excluded two samples (Ctrl_14 and Ctrl_16) from the control group and one sample (E_ASC_7) as an outlier. The count data of the 21 samples were analyzed using the EdgeR 3.36.0. After excluding genes that were not expressed in any of the three sample groups, the count data for 13,292 genes was subjected to TMM normalization. The MDS analysis of the normalized data separated the 21 samples into three clusters: (Group 0 [G0], Group 1 [G1], and Group 2 [G2]) (Figure 4A). All six Ctrl samples were clustered together as Group0. Additionally, E_ASC and L_ASC samples were clustered into either Group 1 or Group 2. Group 1 contained one E_ASC and four L_ASC samples, whereas Group 2 contained six E_ASC and four L_ASC samples. Upon comparing Group 1 and Group 0, we identified 1318 up-regulated genes in Group 1. Those up-regulated genes in Group 1 were enriched with GO terms, such as “Nonsense Mediated Decay independent of the Exon Junction Complex”, “Ribonucleoprotein complex biogenesis”, “Detoxification of Reactive Oxygen Species”, and “Mechanisms associated with pluripotency” (Figure 4B). When comparing Group 2 and Group 0, we identified 1965 up-regulated genes in Group 2. In a gene ontology (GO) analysis using Metascape, the up-regulated genes in Group 2 were found to be enriched with GO terms such as “Nonsense Mediated Decay independent of the Exon junction Complex”, “Detoxification of Reactive Oxygen Species”, “Mechanisms associated with pluripotency”, and “In utero embryonic development.” (Figure 4B). In the comparison of Group 2 and Group 1, we identified 1385 up-regulated genes in Group 2. Those up-regulated genes in Group 2 were enriched with GO terms such as ”Actin cytoskeleton organization”, “Embryonic organ development”, “Embryonic placenta development”, and “Mechanisms associated with pluripotency” (Figure 4B). EVs derived from the mesenchymal stem cells may induce the development of blastocyst, accompanied by a high expression of genes involved in the cell cytoskeleton, pluripotency, and detoxication of reactive oxidative stress, and EVs derived from mesenchymal stem cells with distinct stemness may also generate the development of blastocysts as well as blastocyst outgrowth (Figure 4C).

## 4. Discussion

In the Transwell culture, the fertilized eggs developed into blastocysts using the 3rd-passage stem cells, cultured in the upper layer, more efficiently than those using the 20th-passage stem cells and those without stem cells. In principle, it has been thought that ROS are endogenously generated in pre-implantation embryos as a by-product of ATP synthesis through oxidative phosphorylation and enzymes such as nicotinamide adenine dinucleotide phosphate (NADPH) oxidase and xanthine oxidase and that physiological concentrations of ROS are indispensable for crucial embryonic processes, including pronuclear formation, first cleavage, and cellular proliferation [46,47]. Reportedly, high concentrations of reactive oxygen species induce oxidative stress damage to DNA, proteins, and lipids in cells [48,49,50]. This study observed an upregulation of genes associated with ROS detoxification through GO analysis conducted between the G0 and G1 blastocyst groups, as well as between the G0 and G2 blastocyst groups. As exosomes derived from mesenchymal stem cells can suppress oxidative stress with their antioxidant properties [51,52], the addition of exosomes derived from mesenchymal stem cells may progress the development of blastocysts in the Transwell culture. The developed blastocysts, which were incubated with stem cells, expressed genes associated with pluripotency and embryonic development better than those incubated without stem cells. EVs, including exosomes, which are produced by the 3rd-passage stem cells, participate in the development of blastocysts and possess the characteristics of specific miRNAs. Overall, Transwell culture systems using stem cells are recognized as promising tools for assisted reproductive technology.

In this study, the death of ASCs or fertilized eggs was evident in the culture media. The fertilized eggs were more developed into blastocysts because of the co-culture of ASCs and fertilized eggs in the mixed medium. Under culture conditions, the cell growth of ASCs was stopped. In the Transwell culture system, ASCs normally proliferated, leading to simultaneous significant development in fertilized eggs. In addition, fertilized eggs incubated with 3rd-passage stem cells showed the highest hatching frequency. According to these lines of evidence, blastocysts, which were efficiently developed from fertilized eggs in the Transwell culture system, could be technically feasible and clinically useful for uterine implantation.

The supplementation of IL-6, VEGF, and FGF2 has been documented to be advantageous in the differentiation of fertilized ova [53,54,55]. Additionally, VEGF and FGF are implicated in follicular maturation, as well as in the establishment and sustenance of the corpus luteum [56]. Moreover, IL6, VEGF, and FGF2 have all been demonstrated to facilitate implantation into the endometrium [57,58,59]. Hence, cytokines, such as IL6, VEGF, and FGF2 are frequently touted for their efficacy in enhancing fertility. Nevertheless, studies have reported on the association of elevated IL6 levels in tubal fluids with tubal factor infertility [60], casting uncertainty on its utility. In this study, the paracrine factors, including IL-6 and VEGFA, were not directly associated with blastocyst formation from fertilized eggs. The exosomes extracted from 3rd-passage stem cells significantly induced the formation of blastocysts from fertilized eggs better than those from 20th-passage stem cells. Principal component analysis revealed that exosomes from stem cells induced alterations in the genetic transcripts of all blastocysts. Furthermore, exosomes secreted from the same passage of stem cells did not always indicate constant quality. According to the GO analysis, the introduction of putative high-quality exosomes is involved in nonsense-mediated decay, ribosome assembly, and RNA processing. Furthermore, these genes have been associated with embryonic development [61,62,63,64,65]. In addition, genes associated with pluripotency, ROS detoxification, and embryonic development were highly expressed in blastocysts incubated with stem cells. Compared with the transcripts expressed in blastocysts incubated with stem cells, the genes associated with the actin cytoskeleton, embryonic development, and pluripotency were related to blastocysts introduced with putatively high-quality exosomes. According to these pieces of evidence, exosomes derived from stem cells may play pivotal roles in the maturation and development of fertilized eggs. In vivo, exosomes secreted from tubal epithelial cells are essential factors in the development of fertilized eggs [33]. In in vitro fertilization, the addition of exosomes may contribute to the appropriate embryonic development of fertilized eggs.

Exosomes extracted from 3rd-passage stem cells express specific miRNAs, such as MiR127, which is involved in mesendodermal differentiation during early embryogenesis [66] and placental development [67]. MiR127 may also be associated with ovarian function and testicular function [68,69,70]. Exosomes containing miR127 promoted the differentiation of mouse embryonic fibroblasts into β-like cells with a glucose metabolism function [71,72]. Exosomes harboring miR382 isolated from follicular fluids may play a role in regulating follicle maturation [73]. Additionally, miR382 may participate in the epithelial–mesenchymal transition [74]. It has been reported that miR-181a-2-3p and miR-200c-3p exhibit antioxidant properties [75,76]. As fertilized eggs cultured in vitro are subjected to oxidative stress induced by ROS, the antioxidant properties of these miRNAs may exert a beneficial influence on embryonic development. In this study, the examination of gene expression in exosomes extracted from 3rd-passage stem cells revealed an upregulation in the expression of these miRNAs, which could reinforce this phenomenon. Furthermore, there are many reports concerning miR141-3p, miR676-3p, miR429-3p, miR379-5p, and miR154-5p, which have been associated with the pathogenesis of various cancers and other disorders [77,78,79,80,81]. Therefore, these miRNAs may play promising roles in reproduction and embryonic development.

IL-6 promotes the expression of miRNAs in exosomes [82], which can induce the secretion of IL-6 and IL-8 [83]. Silencing IL-6 significantly inhibits the proliferation of human mesenchymal stem cells. Furthermore, increasing extracellular IL-6 levels cannot restore the proliferative impairment observed in IL-6-silenced human mesenchymal stem cells [84]. In the present study, IL-6 had no effect on blastocyst formation from fertilized eggs. IL-6, VEGFA, and exosomes were better expressed in 3rd-passage stem cells than in 20th-passage stem cells. Based on these findings, it is plausible that IL-6 participates in stemness retention. Unlike VEGF, exosomes promote the crosstalk between inflammation and angiogenesis [85]. However, there are only a few reports suggesting a possible role of VEGF in stimulating exosome production by stem cells. Consequently, IL-6 and VEGF may promote tissue regeneration in the presence of exosomes.

This study demonstrates that exosomes released by ASCs could enhance the differentiation capacity of fertilized ova and characterizes the specific miRNAs that are abundantly present in these exosomes. This study had some limitations. Firstly, the investigation lacked a comprehensive determination of the optimal exosome dosage or the efficacy of individual miRNAs in facilitating ova differentiation. The findings regarding alterations in gene expression in fertilized ova indicate a plausible scenario wherein diverse miRNAs may interact intricately across multiple pathways, warranting elucidation in future research endeavors. Furthermore, the culture technique utilized in this study, employing the Transwell system, is structurally distinct from the conventional culture method currently used in human ARTs. Therefore, when translating the results of this research to human ART techniques, it is challenging to apply the Transwell system directly, necessitating the development of methodologies compatible with actual clinical practice. However, as platelet-rich plasma reportedly plays a role in oocyte maturation and fertilized egg differentiation, the extraction and purification of exosomes using autologous mesenchymal stem cells may render this technology viable for clinical application [86]. Mesenchymal stem cell-derived exosome therapy may have limited clinical application owing to the ethical issues involved. Some countries have approved exosome therapy as long as it is derived from the individual, while others have approved it for exosome therapy derived from others; thus, the views are not uniform. We hope that our research will provide an opportunity to resolve ethical issues.

## 5. Conclusions

In conclusion, in assisted reproductive technologies, the pregnancy rate has been investigated using in vitro-produced embryos. In this study, exosomes secreted from stem cells helped fertilized eggs develop into blastocysts with high hatching potential. Blastocysts express many transcripts that are required for excellent embryonic development. Exosomes from tubal epithelial cells can be inserted into blastocysts during normal pregnancy. Closer to the living body, therefore, the Transwell culture system should be recognized as a beneficial and favorable tool in in vitro fertilization, which may help pregnancy rates in countries with low birth rates. Future studies examining the optimal exosome dosage or the efficacy of individual miRNAs in facilitating ova differentiation may help validate our findings.

## Data Availability

The datasets generated and/or analyzed during the current study are available from the corresponding author upon reasonable request.
